# Examining Public Service Motivation’s Impact on Organizational Commitment: Focusing on Moderating Roles of Hygiene and Motivation Factors

**DOI:** 10.3390/bs14060476

**Published:** 2024-06-05

**Authors:** Geon Jung, Kuk-Kyoung Moon

**Affiliations:** 1 Department of Statistics, Inha University, Incheon 22212, Republic of Korea; starofzurc@gmail.com; 2 Department of Public Administration, Inha University, Incheon 22212, Republic of Korea

**Keywords:** public service motivation, organizational commitment, hygiene factors, motivational factors

## Abstract

Despite previous studies on public service motivation among public sector employees, the empirical analysis of the influential contextual conditions that moderate public service motivation’s impact on employees’ work attitudes remains inadequate. Given these limitations, this study examines public service motivation’s effects on public employees’ organizational commitment and investigates how Herzberg’s hygiene and motivation factors moderate the relationship between these aspects in the context of South Korea’s central government. For this, we used the data of 1021 public employees from the Differences in the Values of Different Generations of Public Officials and Organizational Innovation Survey, which the Korea Institute of Public Administration had conducted in 2022. We analyzed the data through hierarchical multiple regression analyses, and the results indicated that public service motivation exerts a positive effect on organizational commitment. Specifically, hygiene factors weaken the positive relationship between public service motivation and organizational commitment, while motivation factors have a moderating effect that strengthens said relationship. These findings suggest that, in order to enhance organizational commitment among motivated public employees, human resource management practices should prioritize motivational factors that can enhance job content, meaning, and autonomy instead of solely focusing on providing monetary incentives or improving external conditions.

## 1. Introduction

Organizational commitment is essential for the growth and development of public organizations [1], as it plays a crucial role in shaping work-related outcomes for employees (e.g., innovative behavior, job engagement, and turnover intention) and in ensuring organizational stability amid the increasing uncertainties and complexities of the modern administrative environment [2]. Specifically, organizational commitment is crucial for achieving organizational goals and enhancing performance. Public services’ efficacy and efficiency, along with the fulfillment of the public interest, are fundamentally rooted in employees’ deep attachment and commitment to their organizations [3,4]. Such commitment not only fosters a dedicated workforce but also propels the organization toward realizing its objectives, demonstrating its critical importance in the public sector.

In this context, this study conducts an empirical analysis of public service motivation (PSM) as a pivotal determinant of organizational commitment among public sector employees. It is premised on the understanding that job motivation significantly shapes employees’ work attitudes [5]. PSM—defined as a commitment to prioritizing public interest and values over personal gain and contributing positively to the country and its citizens’ welfare—is essential for engaging employees within their organizations [6]. Individuals with a strong inclination toward public service are actively involved in the development of policies aimed at addressing social issues and delivering services that support the public good [7]. This involvement serves to foster a deep sense of belonging and identification with their organizations. As a result, such employees demonstrate a strong commitment to and immersion in both the organization and the policymaking process [8], actively engaging in policy discussions, generating innovative ideas, and sharing information. Moreover, their commitment to the organization’s success and the enhancement of social welfare outweighs their personal interests, leading to a profound loyalty and attachment to the organization as they work toward embedding social values within the public sector [9].

Although the effects of organizational members’ motivations on their work attitudes may vary depending on the organization’s internal and external circumstances and contexts, many previous studies have focused solely on generalized motivational factors without adequately considering these variables [10,11,12]. This oversight has often resulted in a narrow understanding that fails to account for the nuanced ways in which an organization’s unique characteristics can influence the impact of its members’ motivations on work-related outcomes [13]. This research gap underscores the need for further investigation into how different organizational settings, such as varying leadership styles, human-resource-management (HRM) practices, and cultural differences, can either strengthen or impair the relationship between employee motivations and their resulting work attitudes.

Considering the limitations of prior research, the main purpose of this study was to explore the intricate interactions between PSM and organizational commitment within the varying contexts of public organizational environments, as underscored by recent studies [11]. This research was motivated by the increasingly recognized importance not only of the direct impact of PSM on organizational outcomes but also of the contextual factors that can modify these effects [14]. Acknowledging that the organizational context plays a crucial role in shaping the dynamics between PSM and organizational commitment, this investigation meticulously examines the moderating roles of Herzberg’s hygiene and motivation factors. Hygiene factors are directly related to the physical and administrative aspects of the work environment, including the salary, organizational policies, workplace safety, and interpersonal relationships with supervisors [15,16]. In contrast, motivation factors refer to factors that provide intrinsic satisfaction and encompass recognition, responsibility fulfillment, a sense of accomplishment, and opportunities for personal and professional growth [17].

The cognitive evaluation theory specifically explains the moderating effect of hygiene and motivation factors; it states that extrinsic rewards lead to external attribution, thereby weakening individual autonomy and intrinsic motivation [18,19], whereas intrinsic rewards lead to internal attribution by enhancing the meaning and significance of the job’s content [10,20], leading to internal attribution and the enhancement of organizational members’ behavior [21]. From the perspective of the cognitive evaluation theory, the relationship between PSM and organizational commitment can be influenced by both motivational and hygiene factors [19]. More specifically, prioritizing hygiene factors may result in a crowding-out effect, causing employees to perceive external factors as the drivers of their job performance, thus weakening the positive relationship between PSM and organizational commitment [22]. However, the emphasis on motivational factors may lead to a crowding-in effect, which strengthens PSM’s positive effect on organizational commitment by making employees feel more meaningful and autonomous in their jobs [23].

In response to the identified knowledge gaps in the existing literature, this study posed the following research questions: First, does PSM positively influence organizational commitment? Second, do hygiene and motivation factors serve as moderators in the relationship between PSM and organizational commitment? By addressing these questions, we aimed to empirically investigate whether PSM enhances organizational commitment as anticipated. Further, this approach allowed us to assess the efficacy of HRM strategies grounded in Herzberg’s two-factor theory in relation to boosting the organizational commitment of employees driven by PSM. This study also provides practical implications for HRM strategies, specifically examining the effectiveness of HRM practices based on Herzberg’s two-factor theory with respect to strengthening organizational commitment among employees with a high inclination toward public service within public-sector environments. Using this comprehensive approach, this study contributes additional value by providing insights into how targeted HRM interventions can enhance the organizational commitment of highly motivated public-service employees.

The study was conducted as follows: Initially, we examined PSM’s theoretical foundations and reviewed previous studies in this area. Based on this review, we formulated hypotheses regarding PSM’s impact on organizational commitment. Subsequently, we examined the theory that hygiene and motivation factors function as contextual conditions that influence the relationship between PSM and its moderating effects. This examination led to the development of hypotheses concerning the associated moderating effects. Based on this theoretical framework, we have described our research methodology, analyzed the collected data, and interpreted the results. Finally, we have discussed the theoretical and policy implications of our findings.

## 2. Public Service Motivation’s Impact on Organizational Commitment

Organizational commitment is defined as the emotional connection and sense of identification that employees have with their organizations, marked by psychological ties and favorable evaluations between individuals and their organizations [24,25]. Organizational commitment is important in terms of organizational management because employees with high organizational commitment invest their energy and time to achieve organizational goals, and co-operation among team members who share common goals and values enhances their problem-solving abilities, consequently improving the entire organization’s performance and efficiency [26]. In the context of public organizations, which play a crucial role in upholding social values and protecting public interest [27], employees’ commitment is particularly important. Engaged employees are instrumental in improving the quality of public services, effectively implementing public policies, and nurturing public trust and satisfaction [28]. Therefore, uncovering factors that influence employee engagement in public-sector organizations is fundamental to achieving these entities’ objectives and successfully fulfilling their societal roles.

Many previous studies have examined PSM’s impact on organizational commitment, highlighting how employees’ intrinsic drive to achieve organizational goals fosters a sense of attachment and identity within the organization [13,29]. PSM represents a particular type of intrinsic motivation among public sector employees that is characterized by a “concerned for others” type of motivation that prioritizes public interest and values over personal gain. It encapsulates individuals’ dedication to delivering meaningful services to the nation and its citizens, striving for higher-quality public service, and promoting public interest above personal benefits [30,31]. According to a systematic analysis conducted by Perry and Wise [32], this has led to the identification of three core dimensions: rational, normative, and emotional motives. Rational motives mean participating in policy-making with the aim of achieving a change in social norms, while normative motives encompass a commitment to public interest through membership in a public organization and advocacy for public good. Finally, emotional motives are driven by a dedication to protecting the vulnerable and a commitment to societal welfare. (These three categories offer a valuable lens through which PSM can be viewed. However, there is ongoing debate among scholars about whether rational motives constitute a dimension of PSM [33]. In particular, some researchers suggest that rational motives may include self-interested motives [34,35]. The core idea behind rational motives is that individuals make decisions based on a rational evaluation of which option among the several available alternatives will yield maximum utility for them.). These motivations, in particular, define public employees who seek intrinsic fulfillment and value from their work and prioritize the internal satisfaction derived from contributing to public welfare over external rewards [18].

Given PSM’s inherent attributes, it stands to reason that it shares a positive correlation with organizational commitment. First, organizational members who possess strong PSM are driven by personal aspirations to contribute to public well-being through their engagement in public policy [36]. As these individuals deepen their involvement in policy matters, their propensity to actively engage in the formulation and implementation of policies increases [37]. Consequently, it is expected that employees with pronounced PSM will demonstrate a significant degree of attachment and commitment to their organizations. This commitment manifests through their active participation in policy discussions, the creation of innovative proposals, and the dissemination of valuable information, thus reinforcing their organizational identity and dedication to task completion.

From the normative-motivation perspective, employees driven by a strong PSM place a greater emphasis on contributing to their organization’s success and societal welfare than on gaining personal benefits [6]. They establish internal benchmarks that focus on social values that are intrinsic to the public sector and the collective good, leading to an enhanced alignment with the organization, where personal and organizational objectives converge [38]. This alignment deepens their connection with the organization.

Finally, PSM’s emotional dimension further solidifies organizational commitment. Employees who are moved by compassion toward the challenges that disadvantaged or marginalized individuals face tend to strive to impart social value through their dedication and service [39]. Such employees perceive a strong alignment between their values and their professional roles, which culminates in a deeper organizational commitment [40]. This multifaceted relationship between PSM and organizational commitment underscores the profound impact that intrinsic motivations can have on enhancing employees’ dedication and identity within the organization.

Recent studies have continued to substantiate the connection between PSM and organizational commitment, offering compelling empirical support. For example, Sun [41] demonstrated a positive association between PSM and affective commitment among public-sector employees in China. Similarly, Caillier [42] empirically verified the positive influence of PSM on organizational commitment within the U.S. public sector. Further, Kim [43] reported that PSM is a significant determinant of organizational commitment among employees in the Korean government. These findings consistently indicate a favorable link between PSM and organizational commitment across different cultural contexts. Based on these theoretical insights and empirical evidence, the initial hypothesis of this research suggests that a positive relationship exists between PSM and organizational commitment. Hence, we propose the following:

**Hypothesis 1.**  *PSM is positively associated with organizational commitment.*

## 3. Moderating Roles of Hygiene and Motivation Factors

Herzberg’s two-factor theory, which is based on Maslow’s hierarchy of needs, proposes a simplified dual structure of human needs, distinguishing between hygiene and motivation factors, as opposed to Maslow’s layered needs [44,45]. Hygiene factors encompass aspects of the work environment, such as salary, organizational policies, job security, and executive relationships [15,16]. These factors primarily prevent dissatisfaction, and accounting for them avoids discontent but does not enhance satisfaction. The adequate provision of hygiene factors does not necessarily improve work attitudes, while their absence can escalate dissatisfaction, and potentially diminish work motivation [46]. Conversely, motivation factors relate directly to internal satisfaction, including elements such as personal achievement, recognition, responsibility, opportunities for promotion, and personal growth [17]. These factors drive intrinsic motivation, enabling employees to feel fulfilled by their job performance and pursue self-actualization [47]. They are crucial for fostering positive work attitudes and enhancing job performance. The absence of motivation factors may not increase job dissatisfaction, but they do not contribute to creating satisfaction [48]. Herzberg posited that these two sets of factors operate independently, with satisfaction and dissatisfaction forming two separate dimensions rather than being on opposite ends of a single spectrum. This distinction suggests a nuanced approach to managing employee satisfaction and performance by recognizing the unique functions served by hygiene and motivation factors [27].

The contingency theory and previous research suggest that PSM’s impact on organizational performance is contingent on the specific organizational context as well as the characteristics and roles of the organization’s members [8,11,12]. Specifically, an organization’s HRM strategy, which is designed to manage its workforce efficiently, plays a crucial role in either amplifying or hindering motivation’s influence on employees’ work attitudes and behaviors [13]. From this theoretical perspective, motivational factors, which are intrinsic to the tasks that organizational members perform, and hygiene factors, which are extrinsic and pertain to the job’s environmental aspects, could function as contextual moderators. These conditions have the potential to influence how PSM impacts the work attitudes of organizational members.

The moderating effects that hygiene and motivation factors have on the relationship between PSM and organizational commitment can be illustrated using the cognitive evaluation theory formulated by Deci and Ryan [49], which posits that individuals seek to understand the reasons behind their actions by attributing them to various causes, such as external rewards (e.g., salary and bonuses) or environmental factors (e.g., directives and supervision). When an individual attributes their actions to external factors, such as receiving a financial incentive or following explicit instructions, it is viewed as an external attribution [50]. However, if there are no discernible external triggers for their behavior, implying that their actions stemmed from internal satisfaction or interest, it is considered an internal attribution [51,52]. A key aspect of the theory is that offering extrinsic rewards to individuals who are already motivated by internal factors can shift their attribution to an external one. This shift may undermine their sense of autonomy and diminish the impact of their intrinsic motivation. In contrast, enhancing intrinsic rewards can foster a deeper sense of meaning and autonomy through internal attributions, effectively reinforcing behaviors driven by intrinsic motivation [53,54].

The cognitive evaluation theory suggests that PSM’s impact on organizational commitment can be influenced by the presence of motivational and hygiene factors, as PSM inherently embodies intrinsic motivation. When hygiene factors are prioritized, particularly through HRM strategies that emphasize rewards and control, employees may come to view their engagement as being externally driven. This perception can lead to a crowding-out effect, diminishing the strong linkage between PSM and organizational commitment by promoting external attributions over internal ones [22,50]. For example, if an organization excessively focuses on performance-based incentives, detailed supervisory instructions, and stringent control over job performance, individuals with strong PSM might perceive their work as being motivated by external rewards or directives instead of their intrinsic desire to contribute to public welfare [55]. Consequently, such a misalignment could alleviate PSM’s positive impact on organizational commitment.

Conversely, emphasizing motivational factors that enhance job meaningfulness and autonomy can foster intrinsic attributions [19]. HRM strategies that prioritize aspects such as job fulfillment, peer recognition, and opportunities for personal growth can amplify PSM’s positive impact on organizational commitment [19,23]. Employees with high levels of PSM, who aim to enact socially meaningful changes and are dedicated to promoting public good, will have their commitment reinforced if the organization actively supports these intrinsic motivators. This crowding-in effect improves the bond between PSM and organizational commitment by aligning organizational practices with members’ intrinsic motivations. Based on these theoretical arguments, we propose the following hypotheses:

**Hypothesis 2.**  *As hygiene factors increase, the positive relationship between PSM and organizational commitment will weaken.*

**Hypothesis 3.**  *As motivational factors increase, the positive relationship between PSM and organizational commitment will strengthen.*

## 4. Methodology

### 4.1. Data Sources and Sample

The unit of analysis in this study was the individual, and data from the Differences in the Values of Different Generations of Public Officials and Organizational Innovation Survey, conducted by the Korea Institute of Public Administration (KIPA) in 2022, were used to verify the research hypotheses. The data were collected to determine public employees’ generational values in central administrative agencies as well as to identify the generational values’ impact on organizational behavior. The survey period spanned from 24 May to 6 June 2022, and the participants were public employees from central administrative agencies. The survey covered six main areas: professional values, organizational management, personnel management, personal motivation, attitude and behavior, and turnover intention. The KIPA conducted the survey for all 6333 panel members who identified themselves as central government officials. Due to the insufficiency of the existing online panel pool with respect to achieving the desired sample size for this survey, additional data collection was carried out by sending out emails and text messages along with a URL for the survey attached in a co-operation letter from the Ministry of the Interior and Safety (a central government agency that manages government organizations and personnel). To enhance the representativeness of the sample, it was evenly allocated across different age groups. After collecting data from 1046 participants, the responses were validated for sincerity, duplicates were removed, and data cleaning was conducted. Ultimately, out of the total sample of 1021 participants, 714 were received from the online panel pool, while 307 individuals responded through the co-operation letter from the Ministry of the Interior and Safety.

In this study, the items related to the latent variables of PSM, hygiene factors, motivation factors, and job commitment were selected and used by referring to items employed in previous studies. The items were organized on a five-point Likert-type scale (1 = strongly disagree, 2 = disagree, 3 = neither agree nor disagree, 4 = agree, and 5 = strongly agree).

### 4.2. Dependent Variable

This study’s dependent variable was organizational commitment, which refers to a psychological attitude or willingness to strive to achieve the organization’s goals and values continuously and actively through a deep attachment and sense of belonging to the organization [24,25]. To measure organizational commitment, we used four items that imply belongingness, loyalty, and identification: (1) “I feel a strong sense of belonging to my organization”; (2) “I have never thought about leaving my organization”; (3) “The values that my organization pursues are consistent with my values”; and (4) “I am proud to be called a member of my organization”.

### 4.3. Independent Variable

Based on the work of Perry [56], this study measured PSM using four components: attraction to policymaking, public involvement, sympathy, and self-sacrifice. Attraction to policymaking refers to an interest in and favorable attitude toward the formulation and implementation of public policies, alluding to an individual’s willingness to help solve social problems as well as their interest in realizing better social benefits. Commitment to public involvement indicates a commitment and willingness to help advance public interest. Next, sympathy refers to the empathy and compassion felt when the beneficiaries of public services do not receive the appropriate level of benefits. Last, self-sacrifice refers to the extent to which individuals are willing to suffer personal losses for public well-being. Accordingly, four items were used to measure PSM: (1) “Contributing to public good is important to me” (attraction to policymaking); (2) “It is very important to continue to provide administrative services to citizens” (commitment to public involvement); (3) “I feel sorry for people who face difficulties” (sympathy); and (4) “I am ready to make my own sacrifices for society” (self-sacrifice).

### 4.4. Moderating Variables

The moderating variables in this study were motivational and hygiene factors. Motivational factors are intrinsic aspects of the job and include a sense of accomplishment, recognition, and responsibility [57]. The following four items were used to measure motivation factors: (1) “I have enough autonomy to perform my job”; (2) “I am recognized by my colleagues for good job performance”; (3) “My organization provides many opportunities for personal growth”; and (4) “My organization provides promotions that are commensurate with performance”. Hygiene factors are extrinsic aspects of the job and include compensation, work environment, and company policies [57]. The following five items were used to measure hygiene factors: (1) “My compensation is appropriate for my duties and responsibilities”; (2) “My organization’s policies and procedures are reasonable”; (3) “My organization’s employees get along well with each other”; (4) “My organization’s working environment and facilities are good”; and (5) “My organization’s employees receive adequate supervision from my supervisor”.

### 4.5. Controls

Following the research conducted by Bernerth and Aguinis [58], we justified each control variable in our analysis using theoretical frameworks and empirical evidence to demonstrate their potential effects on organizational commitment. Specifically, we incorporated individual background characteristics in our analysis, such as gender, age, tenure, and job grades, as these factors could influence perceived organizational commitment [59,60], although they were not the primary focus of our research. The gender model shows varying levels of commitment, with some studies reporting higher commitment among women [61,62] and others finding higher commitment in men [63,64]. Age is generally positively associated with commitment due to factors such as job satisfaction and fewer employment options for older employees [65,66,67], but some studies report no significant correlation between these two aspects [4,68]. The reported impact of tenure is similarly inconsistent, with some evidence suggesting that it enhances loyalty and emotional attachment, which leads to higher commitment, while other studies indicate no correlation or even a negative relationship between the two [67,68]. The effect of job grade is also debated; some studies report a positive relationship between higher ranks and greater commitment due to increased autonomy and task variety [69,70], while others report no significant effects in this regard [66,71]. These mixed findings highlight the complexity of how individual factors impact organizational commitment, justifying their inclusion as control variables in our analysis to better isolate the specific effects of PSM.

Gender, age, tenure, recruitment method, and rank were included in the analytical model as control variables that may affect organizational commitment. The categorical variable gender was coded as female = 1. Age, an ordinal variable, was coded as 20s = 1, 30s = 2, 40s = 3, and 50s and above = 4. Tenure was coded as less than 10 years = 1, 10–20 years = 2, 20–30 years = 3, and over 30 years = 4. Furthermore, this study categorized job grade as follows: Grades 1–5 = 1; Grades 6–7 = 2; and Grades 8–9 = 3. Table 1 presents the demographic characteristics of the survey respondents.

### 4.6. Measurement Reliability and Validity

Confirmatory factor analyses were conducted to verify the reliability and validity of the newly constructed latent variables. Accordingly, we found that the standardized factor loadings of the survey questions used in this study were above 0.5. The model fit was satisfactory, as the comparative fit index (CFI) and Tucker–Lewis index (TLI) were above 0.9, with a standardized root mean square residual (SRMR) of 0.058, which is below the recommended standard of 0.08. Furthermore, the root mean square error of approximation (RMSEA) had a 90% confidence interval of [0.065, 0.075], thus satisfying the criterion of being 0.08 or less. Finally, the measurement items’ reliability was measured, and each latent variable’s Cronbach’s α value was found to be above 0.7, indicating high reliability (Appendix A).

## 5. Results

Table 2 presents the main variables’ descriptive statistics and the correlations between the latent variables. First, the mean of the main latent variables is 3.04 for the dependent variable and organizational commitment and is 3.55 for the independent variable and PSM. For the control variables, the motivation factor’s mean is 3.32, and the hygiene factor’s mean is 3.14. A statistically significant correlation was found between organizational commitment and PSM (r  = 0.43, *p* < 0.01), motivation factors (r  = 0.59, *p* < 0.01), and hygiene (r  = 0.62, *p* < 0.01). 

Hierarchical multiple linear regression analyses were conducted to test the hypotheses proposed in this study. Hierarchical multiple regression allows for the introduction of independent variables in phases, which is particularly useful for understanding how additional variables influence the relationship between the established predictor(s) and the outcome variable. This approach was chosen to precisely assess how hygiene and motivation factors moderate the influence of PSM on organizational commitment. By adding these variables stepwise, we were able to observe their incremental impact and interaction effects, which was critical for testing our moderation hypotheses.

The results from the analysis are presented in Table 3. (To address potential multicollinearity issues, the mean-centering technique was applied to hygiene factors, motivation factors, and PSM. Following this adjustment, the average variance inflation factor (VIF) scores for the models were reduced to below 2.36. Furthermore, no individual measure’s VIF exceeded 10, demonstrating the absence of significant multicollinearity.). In Model 1, the control variables’ effect on organizational commitment was analyzed, whereas, in Model 2, the direct effect of the independent variable, PSM, on organizational commitment was examined. In Model 3, the moderating effects of motivation and hygiene factors on the relationship between PSM and organizational commitment were determined by entering the interaction terms PSM × hygiene factors and PSM × motivation factors.

In Model 1, the effects of the control variables—gender, age, tenure, and job grade—and the moderating variables—including motivation and hygiene factors—on organizational commitment were analyzed. Among the control variables, gender (β = −0.108, p < 0.01), age (β = −0.099, p < 0.05), tenure (β = 0.167, p < 0.01), and job grade (β = −0.073, p < 0.05) had a statistically significant effect. These results indicate that gender influences organizational commitment among public employees working for the Korean central government. The lower commitment levels observed among female public employees, as compared to their male counterparts, could stem from various factors, including perceived or actual disparities in opportunities for advancement, workplace culture, or work–life-balance-related challenges [73,74]. Moreover, one possible explanation for the negative relationship between age and organizational commitment could be that older employees perceive fewer future career opportunities and feel less valued within the organization. This notion might originate from a bias toward younger employees, who are often seen as more adaptable and technologically adept, potentially leading to older employees feeling disconnected or overlooked in terms of professional development and promotions. In addition, the negative association between job grade and commitment suggests that employees with lower designations may feel a sense of stagnation or limited empowerment, which can significantly diminish their commitment. Employees at lower levels might not only perceive a lack of respect and recognition compared to higher-grade employees but also have fewer opportunities for meaningful engagement or influence within the organization. Finally, the increase in organizational commitment with tenure may reflect the development of stronger ties to the organization over time, increased investment in the organization’s goals, or the receipt of greater benefits and recognition for remaining with the organization longer [75].

Moreover, hygiene (β = 0.378, p < 0.01) and motivation (β = 0.240, p < 0.01) had a statistically significant positive effect on organizational commitment. This suggests that, when organizations effectively address motivation and hygiene factors, it considerably enhances the level of commitment that employees feel toward their organizations. Specifically, motivation factors—which include elements such as recognition, personal achievement, and growth opportunities—directly contribute to enhancing employees’ intrinsic satisfaction and engagement with their work, while hygiene factors—which include aspects such as job security, work conditions, and salary—serve to minimize dissatisfaction. The positive coefficients indicate that improvements in these areas not only reduce employee discontent but also foster a deeper sense of loyalty and dedication toward the organization, thus underscoring the critical function served by these factors with respect to enhancing organizational commitment.

In Model 2, the analysis focused on the influence of the independent variable, i.e., PSM, on organizational commitment. The findings, as anticipated, revealed that PSM (β = 0.148, p < 0.01) positively impacts organizational commitment to a significant extent, thereby confirming Hypothesis 1. This suggests that public employees with a higher degree of PSM tend to exhibit greater levels of commitment to their organization. The intrinsic motivation characteristic of PSM, which aligns one’s personal values and goals with those of their organization, is responsible for this positive effect. Public employees motivated by a desire to promote public well-being and contribute to societal welfare are likely to feel a stronger sense of belonging and loyalty to their organization, particularly if it enables them to fulfill these altruistic goals. This alignment enhances their organizational commitment, as they perceive their work as a means of achieving personally and socially valuable outcomes, thereby validating the initial hypothesis.

In Model 3, the analysis examined the moderating effects of motivation and hygiene factors on the relationship between PSM and job commitment by introducing interaction terms for PSM × hygiene factors and PSM × motivation factors. The findings confirmed the anticipated effects: the positive link between PSM and job commitment was diminished by the hygiene factors (β = −0.100, p < 0.05) and reinforced by the motivation factors (β = 0.159, p < 0.01), hence confirming Hypotheses 2 and 3.

One possible explanation for why hygiene factors weaken the relationship between PSM and organizational commitment lies in the nature of hygiene factors, which include aspects such as job security, salary, and working conditions. These factors, when emphasized excessively, can lead to external attributions for engagement, undermining intrinsic motivations such as PSM. This crowding-out effect suggests that, when employees perceive their involvement as being primarily driven by external rewards or conditions, their intrinsic motivation and, consequently, their commitment to their organizations may diminish.

Conversely, the intrinsic nature of motivation factors explains why they strengthen the relationship between PSM and organizational commitment. Motivation factors, such as recognition, personal achievement, and opportunities for growth, align closely with PSM. When employees view their work as an avenue for personal and professional growth and recognition, their intrinsic motivation is amplified, leading to increased organizational commitment. This crowding-in effect indicates that enhancing intrinsic rewards and job content that align with employees’ PSM can significantly bolster their commitment to the organization.

**Figure 1:**
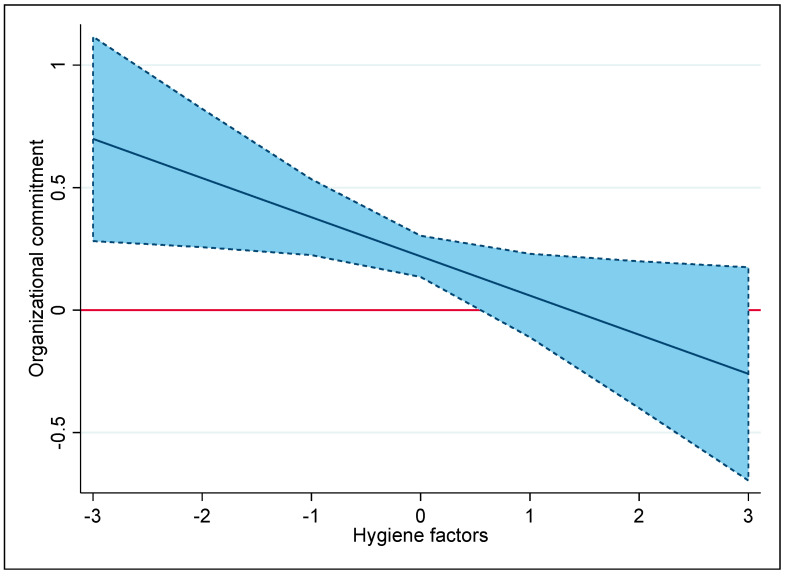
Marginal effect of PSM on organizational commitment under varying levels of hygiene factors.

Figure 1 illustrates in more detail the marginal effect of PSM on organizational commitment with changes in hygiene factors. The blue area represents the 95% confidence interval for these predictions. The area above and below the horizontal zero line implies that the effect of hygiene factors is statistically significant. The figure confirms that the positive marginal effect of PSM declines as the number of hygiene factors increases. Once hygiene factors near 1 (high levels of hygiene factors), PSM no longer has a significant effect on public employees’ organizational commitment. This finding is consistent with evidence suggesting that hygiene factors hinder the positive relationship between PSM and organizational commitment. Specifically, when hygiene factors are emphasized, especially through HRM strategies that focus on rewards and control, employees might perceive their involvement as being externally motivated. This can trigger a crowding-out effect, impairing the positive connection between PSM and organizational commitment by leading employees to favor external motivations over internal ones.

**Figure 2:**
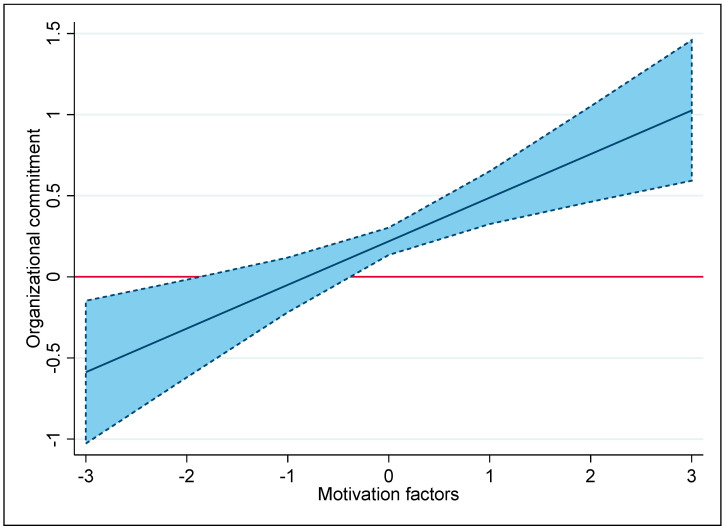
Marginal effect of PSM on organizational commitment under varying levels of motivation factors.

Figure 2 vividly portrays how varying levels of motivation factors positively influence the effect that PSM has on organizational commitment. The graph shows that, when motivation factors, such as responsibility, recognition, and personal-growth opportunities, increase, they significantly boost the impact of PSM on organizational commitment. Once motivation factors exceed 0 (medium levels of motivation factors), the relationship between PSM and organizational commitment is bolstered. This finding implies that employees who possess a strong sense of PSM, i.e., who are committed to making socially impactful changes and are dedicated to public welfare, will experience a boost in their commitment if their organizations actively nurture these intrinsic motivators. This crowding-in effect solidifies the link between PSM and organizational commitment by ensuring that organizational practices are in harmony with the intrinsic motivations of its members.

## 6. Discussion

This study investigated PSM’s influence on organizational commitment in the public sector by determining how hygiene and motivation factors moderate this relationship. By integrating situational and cognitive evaluation theories, it analyzed how these factors modify PSM’s impact. The results indicated that a high degree of PSM leads to increased organizational commitment, thereby supporting Hypothesis 1. Furthermore, the study found that hygiene factors reduce the positive effect that PSM has on organizational commitment, thus supporting Hypothesis 2. Conversely, motivation factors were found to enhance this positive impact, which confirms Hypothesis 3. These findings highlight the distinct roles played by hygiene and motivation factors in shaping the relationship between PSM and organizational commitment.

Our findings confirm the findings reported in the literature, demonstrating a positive association between PSM and organizational commitment in public organizations. This result is consistent with previous studies that revealed a positive association between PSM and organizational commitment [13,41,76]. This alignment not only corroborates previous studies but also enriches the theoretical framework by elucidating the mechanisms through which employees with high PSM integrate their personal ambitions with their organization’s broader social and public-interest-related goals, which primarily involves active public-policy engagement. This integration strengthens the connection between employees and their organizations significantly, with intrinsic and “other-concerned” motivation playing essential roles in enhancing this bond. By detailing how these motivational dimensions interact to bolster organizational commitment, our study provides a nuanced understanding of the underlying dynamics. This contribution is vital, as it not only reaffirms the positive relationship between PSM and organizational commitment but also offers a comprehensive explanation of the mechanisms underpinning this link. These can be of great benefit, as they advance theoretical knowledge and inform practical approaches in public management.

Our research significantly forwards the discourse on PSM by delineating the distinct impacts of hygiene and motivational factors on organizational commitment within the framework of the cognitive evaluation theory. While existing studies on the crowding-out and crowding-in effect provide mixed and inconclusive results [19,23], our findings offer a clearer perspective. We demonstrate that, while hygiene factors can undermine the impact of PSM by shifting employees’ focus to external rewards—effectively weakening their intrinsic motivation and organizational commitment—motivational factors can enhance this commitment by reinforcing intrinsic motivations.

This study supports the notion that motivational factors induce a crowding-in effect, which enhances the bond between PSM and organizational commitment by promoting a sense of internal control. In contrast, the crowding-out effect observed with hygiene factors highlights the detrimental impact of extrinsic rewards on intrinsic motivations—which are crucial for employees engaged in public service—that occurs through the creation of a sense of external control. These insights represent a robust application of the cognitive evaluation theory to public-service settings and suggest that striking a balance between extrinsic and intrinsic motivations is the key to fostering robust organizational commitment.

Theoretically, this research enriches our understanding of how different types of motivation interact within the framework of public service. It challenges the simplistic binary perspective of intrinsic versus extrinsic motivation by demonstrating that the impact of these motivations on organizational commitment is mediated by specific organizational practices and policies. This insight serves to enhance the theoretical discussions regarding motivation in the public sector by suggesting that the effects of motivational strategies are contingent on their alignment with employees’ intrinsic values and HR strategies. Further, from an academic standpoint, these findings contribute to a more refined understanding of employee behavior in the public sector, providing empirical evidence that supports a novel approach to motivational strategies. This contributes to a broader discourse on public administration by suggesting that public-sector organizations can enhance employee commitment and performance by not only providing financial incentives but also strategically fostering environments that amplify the positive effects of PSM.

Based on this study’s findings, we propose several practical implications. Given that hygiene factors do have a positive effect on organizational commitment, organizations need to manage these factors appropriately. For example, the effective management of hygiene factors—including workplace safety, a fair salary structure, supervisory management and control, and clear rules and procedures—can prevent unnecessary stress and dissatisfaction among employees, thus fostering a sense of belonging and commitment to the organization. However, an excessive emphasis on hygiene factors can cause employees to focus on simply avoiding dissatisfaction and, most importantly, can weaken the positive relationship between PSM and organizational commitment. Hence, while benefits, salary, remuneration, and job security are certainly important factors, these hygiene factors alone are unlikely to improve the meaningfulness and satisfaction that employees with PSM experience with regard to their work. Therefore, if public organizations want to increase their employees’ commitment and attachment to them, they should improve their HRM strategies in terms of motivational factors, such as employees’ sense of accomplishment, responsibility, and security as well as their work’s importance.

To enhance the organizational commitment of employees, especially those with high PSM, public organizations need to implement multifaceted strategies that encourage both personal and professional growth. First and foremost, it is crucial that they provide ample opportunities for career advancement. This could be appropriately addressed through robust personal development programs that include mentoring, professional training, workshops, and clear avenues for promotion and internal mobility. Such initiatives could help employees envision a future filled with growth potential within their organizations and, as a result, enhance their engagement with and commitment to them.

In addition, the importance and diversity of each job role should be consistently reinforced. Regular job reviews and team meetings should be conducted to discuss and analyze how each public employee’s efforts contribute to the overarching goals of the organization. During these discussions, it is vital to highlight the direct consequences of the employees’ work for the organization’s performance, thereby developing a deeper connection between individual contributions and organizational success. This approach could not only increase the employees’ sense of organizational attachment but also instill a sense of pride in them with respect to their contributions.

Moreover, implementing a well-defined succession-planning system that identifies potential future leaders and prepares them through targeted development plans could ensure organizational continuity and motivate employees by providing them with a clear career trajectory. This strategy would not only reinforce their commitment but also reduce any uncertainty they may feel about future opportunities. Furthermore, it is crucial to integrate elements of PSM into these career development frameworks. By creating roles or career paths specifically designed to have a direct impact on public-service outcomes, organizations could effectively align employee aspirations with public-sector values. Such alignment could enhance organizational commitment, as it would enable employees to see a tangible connection between their career progression and the broader goals of public service, thus enhancing their engagement with and dedication to the organization’s mission.

Finally, it is essential to increase the autonomy and responsibility given to employees with high PSM to deepen their organizational commitment. While public organizations should set clear goals and expectations for their employees, they should allow them the freedom to determine how they would like to achieve these objectives. Such autonomy serves to empower employees and enhance their job satisfaction. Regular performance reviews and personalized feedback are critical in this structure, as these help highlight individual achievements and hold employees accountable for their outcomes. By implementing these suggestions, public organizations should be able to motivate their employees to explore creative solutions, set ambitious goals, and develop a more profound understanding of their roles.

Despite the theoretical and practical implications of this study, it has certain limitations. First, the cross-sectional data used in this study cannot reflect temporal changes, which limited our ability to identify correlations between independent and dependent variables as well as the generalizability of the findings. To overcome this, future studies should utilize longitudinal data or combine various research methods, such as in-depth interviews and experimental designs, to strengthen the correlations’ validity. Further, we utilized secondary data provided by the KIPA to test our hypotheses, which implies that the questionnaire used in the study may not fully reflect the complex constructs of PSM, organizational commitment, and motivation and hygiene factors, which are the study’s core variables. Therefore, future researchers should develop a questionnaire tailored to the study’s purpose and use the collected data to measure and analyze the study’s core variables more accurately. Second, it is evident that the study has certain limitations regarding the measurement of latent variables. Future research should focus on developing and refining items that accurately measure each latent variable to ensure a clearer and more precise assessment. In addition, increasing the sample size could enhance both convergent and discriminant validity. By addressing these aspects, future studies can provide enhanced validation of the constructs and potentially offer more definitive findings that meaningfully contribute to the field. Finally, in this study, we employed organizational commitment as the dependent variable to assess the effects of PSM. However, many previous studies have utilized organizational commitment as a mediating variable to measure both direct and indirect effects on various work-related outcomes. Therefore, to extend the research on PSM and organizational commitment, it is necessary to use organizational commitment as a mediator to further assess its effects.

## 7. Conclusions

PSM plays a crucial role in enhancing organizational commitment among public-sector employees. Importantly, this effect is moderated by both hygiene and motivation factors, as delineated by Herzberg’s two-factor theory. We found that, while hygiene factors are essential for preventing dissatisfaction within the workplace, their overemphasis, particularly when tied to external rewards and controls, can diminish the positive effects of PSM. This is due to a crowding-out effect, where external motivations undermine intrinsic ones, leading to reduced organizational commitment. Conversely, motivation factors have a crowding-in effect that enhances the positive impact of PSM on organizational commitment. When public organizations focus on improving job content, meaning, and autonomy, they not only meet the intrinsic needs of their employees but also align these enhancements with the employees’ PSMs, thus strengthening their commitment.

This study emphasizes the need for well-designed HRM that is based on motivating rather than hygienic HR practices. Public organizations seeking to maximize the organizational commitment of staff must create an environment that magnifies motivation factors, making their employees feel authentic purpose and engagement in their roles. These results contribute to an improved comprehension of the relationship between PSM and organizational commitment and offer practical implications for HRM in public organizations. Further research is required to investigate this complex relationship between PSM, hygiene and motivation factors, and organizational commitment so that the current insights can be built upon to improve organizational strategies.

## Figures and Tables

**Table 1:** Demographic information of the survey respondents.

Background	Category	Number of Cases	Percentage (%)
Gender	Female	562	55.04
	Male	459	44.96
Age	≤29	226	22.14
	30–39	318	31.15
	40–49	305	29.87
	≥50	172	16.85
Tenure	≤9	539	52.79
	10–19	279	26.93
	20–29	156	15.28
	≥30	51	5.00
Job grade	Grades 1–5	343	33.59
	Grades 6–7	499	48.87
	Grades 8–9	179	17.53

**Table 2:** Descriptive statistics and correlations.

	(1)	(2)	(3)	(4)	(5)	(6)	(7)	(8)
(1)	1							
(2)	0.43 **(0.30)**	1						
(3)	0.59 **(0.57)**	0.45 **(0.33)**	1					
(4)	0.62 **(0.59)**	0.42 **(0.90)**	0.74 **(0.28)**	1				
(5)	−0.13	−0.13	−0.04 †	−0.05 †	1			
(6)	0.10	0.18	−0.01 †	0.01 †	−0.33	1		
(7)	0.15	0.18	0.06 †	0.09	−0.25	0.77	1	
(8)	−0.13	−0.16	−0.14	−0.14	0.21	−0.48	0.53	1
Mean	3.04	3.55	3.32	3.14	1.45	2.41	1.78	6.86
S.D.	0.85	0.62	0.61	0.69	0.50	1.01	0.93	1.42
AVE	0.63	0.51	0.48	0.42	-	-	-	-

Note: (1) Organizational commitment; (2) PSM; (3) motivation factors; (4) hygiene factors; (5) gender; (6) age; (7) tenure; (8) job grade; † = not significant at 95% confidence interval; S.D. = standard deviation; AVE = average variance extracted; the numbers written in bold in parentheses represent the squared correlations. (For convergent validity, the AVE should be equal to or greater than 0.5, whereas, for discriminant validity, it should exceed the squared correlations among the dimensions. First, the AVE values of organizational commitment and PSM were above 0.5, indicating acceptable measurement reliability. However, the AVE values for the motivation and hygiene factors were below 0.5, which implies a lower convergent validity for these factors according to Fornell and Larcker (1981). Nevertheless, this criterion was recognized as conservative. Considering that the AVE values were close to the threshold and that they aligned with theoretical expectations, the slightly lower AVE values were not of significant concern. Further, it is acceptable for AVE values to be below 0.5 if the construct reliability exceeds 0.7 [72]. Second, for some latent variables, the AVEs were less than the squared correlations of these variables with other constructs, indicating mixed evidence for discriminant validity, which was expected given the imprecisely defined four-factor structure).

**Table 3:** Hierarchical multiple linear regression models for the hypothesized relationships.

	Model 1		Model 2		Model 3	
	β (SE)		β (SE)		β (SE)	
Gender (female = 1)	−0.108	***	−0.067	***	−0.065	***
	(0.057)		(0.042)		(0.042)	
Age	−0.099	**	0.006		−0.001	
	(0.044)		(0.032)		(0.033)	
Tenure	0.167	***	0.068	*	0.071	*
	(0.046)		(0.036)		(0.036)	
Job grade	−0.073	**	0.025		0.023	
	(0.045)		(0.034)		(0.034)	
Hygiene factors			0.378	***	0.370	
			(0.053)		(0.052)	
Motivation factors			0.240	***	0.234	
			(0.055)		(0.054)	
PSM			0.148	***	0.160	**
			(0.043)		(0.043)	
PSM × Hygiene factors					−0.100	**
					(0.071)	
PSM × Motivation factors					0.159	***
					(0.073)	
$R^{2}$	0.040		0.457		0.466	

Note: * *p* < 0.1; ** *p* < 0.05; *** *p* < 0.01; β = standardized coefficient; SE = robust standard error; and N = 1021.

## Data Availability

The data used for this study are available at https://www.kipa.re.kr/site/kipa/stadb/selectBaseDBFList.do, accessed on 8 February 2024. Permission to use the data must be obtained from the KIPA.

## Appendix A. Factor Loadings and Cronbach’s α for All Latent Variables

**Latent Variables**	**Survey Items**	**Factor Loading**	**Cronbach’s α**
Organizational commitment	▪ I feel a strong sense of belonging to my organization.	0.81	0.86
	▪ I have never thought about leaving my organization.	0.65	
	▪ The values that my organization pursues are consistent with my values.	0.83	
	▪ I am proud to be called a member of my organization.	0.86	
Public service motivation	▪ Contributing to public well-being is important to me.	0.79	0.80
	▪ It is very important to continue to provide administrative services to citizens.	0.75	
	▪ I feel sorry for people who face difficulties.	0.63	
	▪ I am ready to make my own sacrifices for society.	0.69	
Motivation factors	▪ I have enough autonomy to perform my job.	0.58	0.74
	▪ I am recognized by my colleagues for good job performance.	0.54	
	▪ My organization provides many opportunities for personal growth.	0.74	
	▪ My organization provides promotions that are commensurate with performance.	0.71	
Hygiene factors	▪ My compensation is appropriate for my duties and responsibilities.	0.62	0.81
	▪ My organization’s policies and procedures are reasonable.	0.80	
	▪ My organization’s employees get along well with each other.	0.63	
	▪ My organization’s working environment and facilities are good.	0.64	
	▪ My organization’s employees receive adequate supervision from my supervisor.	0.75	
